# Do less populous countries receive more development assistance for health per capita? Longitudinal evidence for 143 countries, 1990–2014

**DOI:** 10.1136/bmjgh-2017-000528

**Published:** 2018-01-03

**Authors:** Lene Martinsen, Trygve Ottersen, Joseph L Dieleman, Philipp Hessel, Jonas Minet Kinge, Vegard Skirbekk

**Affiliations:** 1 Department of International Health, Norwegian Institute of Public Health, Oslo, Norway; 2 Institute for Health Metrics and Evaluation (IHME), University of Washington, Seattle, USA; 3 Alberto Lleras Camargo School of Government, University of the Andes, Bogotá, Colombia; 4 Department of Epidemiology, Norwegian Institute of Public Health, Oslo, Norway; 5 Centre for Fertility and Health, Norwegian Institute of Public Health, Oslo, Norway

**Keywords:** health economics, health policy, public health

## Abstract

**Background:**

Per capita allocation of overall development assistance has been shown to be biased towards countries with lower population size, meaning funders tend to provide proportionally less development assistance to countries with large populations. Individuals that happen to be part of large populations therefore tend to receive less assistance. However, no study has investigated whether this is also true regarding development assistance for health. We examined whether this so-called ‘small-country bias’ exists in the health aid sector.

**Methods:**

We analysed the effect of a country’s population size on the receipt of development assistance for health per capita (in 2015 US$) among 143 countries over the period 1990–2014. Explanatory variables shown to be associated with receipt of development assistance for health were included: gross domestic product per capita, burden of disease, under-5 mortality rate, maternal mortality ratio, vaccination coverage (diphtheria, tetanus and pertussis) and fertility rate. We used the within-between regression analysis, popularised by Mundluck, as well as a number of robustness tests, including ordinary least squares, random-effects and fixed-effects regressions.

**Results:**

Our results suggest there exists significant negative effect of population size on the amount of development assistance for health per capita countries received. According to the within-between estimator, a 1% larger population size is associated with a 0.4% lower per capita development assistance for health between countries (−0.37, 95% CI −0.45 to –0.28), and 2.3% lower per capita development assistance for health within countries (−2.29, 95% CI −3.86 to –0.72).

**Conclusions:**

Our findings support the hypothesis that small-country bias exists within international health aid, as has been previously documented for aid in general. In a rapidly changing landscape of global health and development, the inclusion of population size in allocation decisions should be challenged on the basis of equitable access to healthcare and health aid effectiveness.

**Key questions**

**What is already known about this topic?**

- Previous studies have identified that development assistance per capita decreases with population size of the receiving country.
- It has been hypothesised that a similar pattern exists for development assistance for health.
- However, although a number of studies on health aid include population size as a control variable, we found no studies addressing this hypothesis specifically.

**What are the new findings?**

- Our analysis provides evidence that there exists a negative association between population size of recipient countries and the amount of development assistance for health per capita countries receive.
- The association exists when comparing across countries and when assessing population growth across time within a country.
- This implies that individuals being part of smaller populations are favoured with regard to global health aid funding allocations.

**Recommendations for policy**

- No funding agency officially argues that individual need in countries with larger population sizes is less important than in countries with lower population sizes. Yet, health-related aid is allocated disproportionately to countries with lower population sizes, revealing a discrepancy in the aid allocation process.
- Donors should be informed that individuals residing in countries with smaller populations are given priority—and a debate on whether this is a correct allocation should be encouraged.
- New allocation criteria should consider what criteria promote the most fair and effective allocation, including whether lower significance given to those in need residing in countries with greater population sizes is justified.

## Introduction

The total amount of development assistance for health (DAH) allocated has increased steadily since 1990^1 2^ to US$37.6 billion in 2016.^3^ A critical, yet often neglected factor for funders of development assistance is how population size of recipient countries should influence their allocation decisions.

From the perspective of an individual’s rights and the view that every person has an equal right for coverage of basic health needs, it is plausible that the amount of support each individual receives should be independent of the size of the population of which she or he is part, unless economy of scale compensates for this (ie, the health systems in these countries are able to treat a larger number of patients for the same cost as in countries with smaller populations). Following this rationale, population size per se should not influence the per capita allocations of health aid when adjusted for needs.

However, some donors have explicit allocation practices that are at odds with the principle that population size should not affect individual allocations.^4–6^ For example, the allocation formula used by the United Nations (UN) Development Programme (UNDP), where population has been a key parameter in the programming arrangements since 1995 and increasing population size results in lower aid per capita. The World Bank’s (WB) focus on small states (states with population size of <1.5 million people)^7^ is another example of health aid allocation with the attention on less populous countries. Further, studies have shown that overall development assistance (ie, development assistance to health and all other areas) per capita is negatively associated with the population size of recipient countries,^8–13^ which means that many funders implicitly give more aid per capita to less populous countries.

This ‘small country effect’ was first mentioned by the Organisation of Economic Co-Operation and Development (OECD) in 1969^14^ that suggested that smaller nations had a greater need of aid to finance imports compared with larger nations. We further consider a number of potential rationales for giving more DAH per capita (DAHpc) to countries with lower population sizes in the interpretation of our results in the Discussion section. These rationales are related to economy of scale, accountability, funders’ international influence as well as recipients’ visibility and vulnerability.

Although population size has been included as a control variable in a few studies about the allocation of DAHpc,^15 16^ no study has systematically assessed the effects of increases in population size on the receipt of DAHpc using longitudinal data for a large number of countries. Because a number of middle-income countries (MICs) have a rapid population growth,^17–19^ these countries could potentially be given less importance when aid allocation decisions are made both because of their economic development (which could make them seen as less dependent on economic transfers) as well as their large population size (see Discussion section). This is despite the fact that the majority of the world’s poor are inhabitants of these countries and that their burden of disease is often relatively high.^20^ Against this background, the objective of this study was to systematically assess the relationship between population size and DAHpc using data for 143 countries over the period 1990–2014.

## Methods

### Data sources

Information on DAH came from Institute of Health Metrics and Evaluation (IHME) database.^3^ The IHME database covers DAH, and includes public as well as private sources and adjusts for double counting, that is, when donors channel aid through multilateral agencies.^2 21^

Information on population size and covariates came from the WB, IHME and the UN databases. Burden of disease data for 1990–2015 was retrieved from IHME. Data on fertility levels came from UN’s Population Division, which contains key demographic indicators for each country for the years 1950–2015.^22^ The remaining covariates came from the WB Open Data Initiative.^23^ Table 1 shows all variables including sources.

**Table 1:** Definitions of variables

Variable name	Description	Source
DAHpc	Development assistance for health per capita in 2015 US$	IHME
POP	Total population size in recipient country	WB
GDPpc	Gross domestic product per capita in current US$	WB
DALYR	Burden of disease measured as disability-adjusted life years (rate)	IHME
U5MR	Mortality rate, under-5 (per 1000 live births)	WB
MMR	Maternal mortality ratio (modelled estimate, per 1 00 000 live births)	WB
DTP3	Immunisation, diphtheria, tetanus and pertussis (% of children aged 12–23 months who received vaccination)^48^	IHME
TFR	Total fertility rate (children per woman)	UN

IHME, Institute for Health Metrics and Evaluations; UN, United Nations; WB, World Bank.

The study covered the period 1990–2014, as information on health-related development was available from IHME for those years. Countries were considered sample eligible if they were LICs or MICs in 1990, as defined by the WB, received health-related development assistance for 5 years or more between the years 1990 and 2014, and if data on covariates for those countries and years were available. As some recipients of DAH are overseas territories of donor countries, for example, Anguilla, French Guiana and Cook Island, limited data were available for those territories. Total number of countries fulfilling these criteria was 143, resulting in a total of 3572 of DAHpc (see online supplementary appendix table A1 for details).

10.1136/bmjgh-2017-000528.supp1 Supplementary file 1

### Measures

#### Development assistance for health

The dependent variable for this study was total DAHpc of recipient countries. DAH was defined as the amount of financial and in-kind assistance that is tracked from source to channel to recipient country and health focus area (IHME, 2016). DAHpc was measured in constant 2015 US dollars.

#### Population size

The main independent variable of interest was total population size of recipient countries.

#### Covariates

We included several additional explanatory variables that have been shown to be associated with receipt of DAH,^21 24–26^ including: (1) gross domestic product per capita (GDPpc), (2) overall burden of disease in terms of rate of disability-adjusted life years (DALYR), (3) under-5 mortality rate (U5MR), (4) maternal mortality ratio (MMR), (5) vaccination coverage represented by diphtheria, tetanus and pertussis (DTP3) immunisation and (6) total fertility rate (TFR). TFR was included because it is tightly linked to population size. Prevalence of human immunodeficiency virus (HIVprev) has been a major driver of health-related development assistance but was not included as a covariate because data on HIVprev do not cover countries with population sizes <250 000 and several of the countries included in our analyses have population sizes below that threshold.^27^ However, we included HIVprev as a covariate in the robustness checks.

We explored a number of additional covariates that might affect the allocation of DAH to test the robustness of the model (see online supplementary appendix). These covariates were: HIV prevalence, life expectancy at birth, water access, surface area (km^2^), freedom of expression (the voice and accountability index from Worldwide Governance indicator) and conflict existence (the political stability and absence of violence index from Worldwide Governance indicator). However, none of these covariates changed the results, hence, they were not included in the main regression analysis.

Age structure of the populations was considered a potential confounder since health-related aid has been focused on areas affecting the younger segment of populations during the Millennium Development Goals (eg, reducing child and maternal mortality, reducing childhood stunting and the need of modern contraception).^28 29^ Therefore, the percentage of the population between those aged 0 and 14 years was initially included in the analyses, but subsequently omitted due to strong multicollinearity.

Since burden of disease data is only estimated for 5-year intervals (1990, 1995, 2000, 2005, 2010 and 2015), we used log-linear interpolation between the estimates to calculate yearly data (constant per cent change per year).

All variables, except fertility and DTP3, were log transformed due to right-skewedness in their distributions (see online supplementary appendix).

### Statistical analyses

To examine the relationship between DAHpc and population size of the recipient countries, we used the within-between estimator^30^ to conduct a regression of panel data covering the years 1990–2014. This analysis assess whether changes in population size over time are associated with changes in DAH a country receives, and whether the difference in population size between countries is influencing the allocation of DAHpc between the different countries. The rationale for this approach was that the within-between estimator controls for time-invariant unobserved heterogeneity assessing the variation across time (ie, within-country estimates) while also measuring across country effects by adding group means for the independent variables (ie, between-country estimates).

In addition, we included three alternative regressions as robustness checks. An OLS (ordinary least squares) cross-sectional analyses (1) covering only the most recent 5-year period (the average of 2010–2014). This analysis shows a snapshot of the currently existing association between DAHpc and population size. We also used random-effects (RE) (2) as well as fixed-effects (FE) (3) models for the full panel data set (1990–2014). Country-clustered robust SEs were used in all the primary analysis as well as the RE and FE regression models.

Since all variables except fertility and DTP3 have a strongly skewed distribution, we naturally log-transformed (ln) the variables to approximate a normal distribution (see online supplementary appendix). The coefficients in a log-log model represent the *elasticity* of the dependent variable with respect to the independent variables. In other words, the coefficient is the estimated *per cent change* in the dependent variable for a *per cent change* in the independent variable.

We also assessed the existence of a non-linear relationship between DAHpc and population size by using splines. However, the association between (log) DAHpc and (log) population size did not show any non-linear patterns.

The within-between model can be written as:

$$\begin{array}{ll} DAHpc_{it}= & \alpha_{i}+\beta_{1w}P\dot{O}P_{it}+\beta_{1b}{\bar{POP}}_{i}+\beta_{2w}{\dot{X}}_{it}\\ & +\beta_{2b}{\bar{X}}_{i}+\beta_{3}YEAR_{t}+\varepsilon_{it} \end{array}$$

Where,

$${\dot{X}}_{it}\equiv X_{it}-{\bar{X}}_{i}\;(\bar{X}=mean\;\;of\;\;X)$$

$DAHpc_{it}$ is the natural log of health-related development assistance per capita in country *i* at time *t*, $POP_{it-1}$ is natural log of the population size at time *t,* $X_{it}$ is a vector of covariates for country *i* at time *t* (see online supplementary appendix for details), α is the mean country random effects, $\beta_{3}YEAR_{t}$ is time dummies and $\varepsilon_{it}$ the error term.

The within-between regression was also done without the covariates (ie, only including population size) (see online supplementary appendix).

The specifications for the OLS, RE and FE models are given in the online supplementary appendix.

## Results

The amount of DAHpc ranged from US$0 to US$359 during the years 1990–2014. The population size of recipient countries ranged from 74 000 to 1.36 billion people. Summary statistics for all the variables can be found in the online supplementary appendix.

Table 2 shows results from the within-between estimator for the years 1990–2014. The coefficient estimates associated with the within-country means measure the between-country association between each covariate and development assistance, while the coefficient estimates for the time-varying covariates (which have country means removed), measure the within-country association between each covariate and development assistance. The results suggest that 1% larger population size based on the between-population variation (lnPOP_mean) is significantly associated with a 0.37% lower DAHpc ($\beta_{1b}$=−0.37, 95% CI −0.45 to –0.28), while 1% larger population size based on the within-population variation (lnPOP_demean) is significantly associated with a 2.29% lower DAHpc ($\beta_{1w}$=−2.29, 95% CI −3.86 to –0.72). GDPpc is significantly associated with DAHpc for the between-population coefficient but not for the within-population coefficient suggesting that economically poorer countries receive more DAHpc than economically richer countries but the economic changes within a country are not significantly associated with changes in DAHpc. The DALY rate is not significant for neither the within nor between coefficients. The MMR is positively associated with DAHpc for the between-country coefficient, that is, higher rates of maternal mortality leads to more DAHpc, but is not significant for the within-country coefficient. The immunisation covariate (percentage of children under the age of 12 years that has received a DTP3 vaccine) is positively significant for only the within-population coefficients. Fertility is not significantly associated with DAHpc for neither the within nor the between coefficients.

**Table 2:** Summary of the regression analysis using the within-between estimator

Covariate	Coefficient	P value	95% CI
lnPOP_mean	−0.37	0.00	−0.45 to −0.28
lnPOP_demean	−2.29	0.00	−3.86 to −0.72
lnGDPpc_mean	−0.26	0.02	−0.49 to −0.04
lnGDPpc_demean	−0.21	0.29	−0.60 to 0.18
lnDALYR_mean	−0.23	0.53	−0.96 to 0.49
lnDALYR_demean	−0.03	0.97	−1.22 to 1.16
lnU5MR_mean	0.10	0.73	−0.45 to 0.64
lnU5MR_demean	0.42	0.36	−0.47 to 1.31
lnMMR_mean	0.63	0.00	0.28 to 0.97
lnMMR_demean	−0.56	0.17	−1.35 to 0.24
DTP3_mean	0.30	0.59	−0.80 to 1.41
DTP3_demean	0.01	0.07	0.00 to 0.02
fertility_mean	−0.07	0.45	−0.26 to 0.12
fertility_demean	−0.11	0.41	−0.37 to 0.15
Number of observations	3475		
Number of countries	143		

The outcome variable is the natural log of DAHpc. The mean covariates gives the between-group (country) effect, while the demean covariates gives the within-group (country) effect.

**Figure 1:**
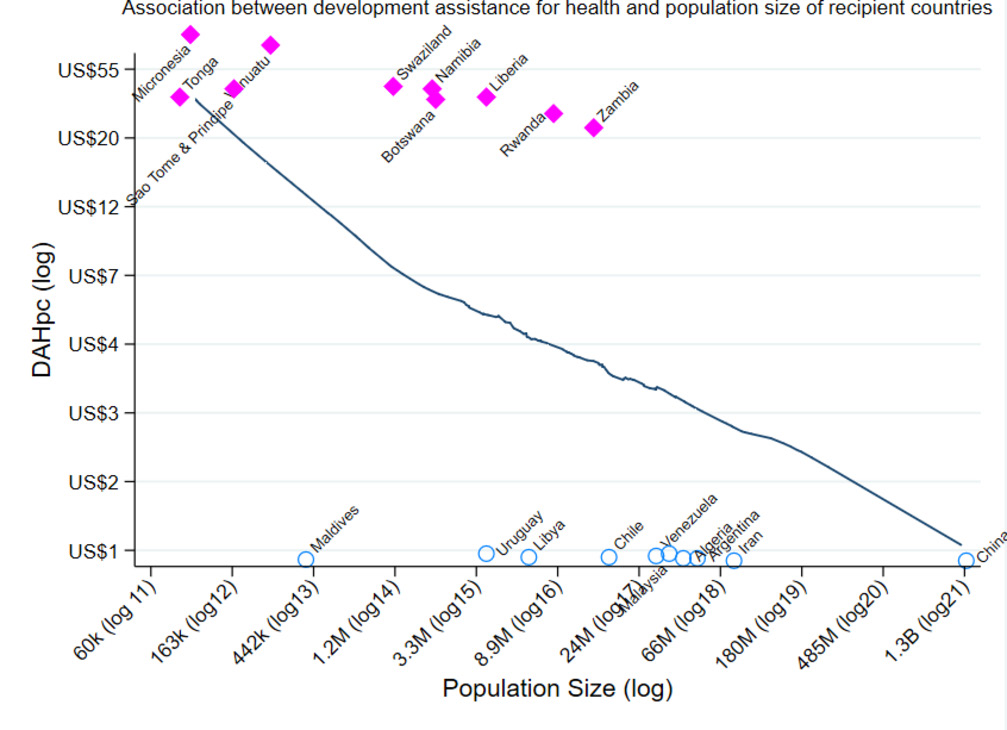
The association of development assistance for health per capita (DAHpc) and population size (POP). The graph is a locally weighted regression of logDAHpc and logPOP. The axis show the actual values of DAHpc in US dollars (US$), and POP in thousands (K), millions (M) and billions (B) with the log value in brackets. This graph is based on the pooled data for all the years 1990–2014. The correlation coefficient for DAHpc (ln) and population size (ln) is −0.31. Pink, square dots show the 10 countries receiving the highest amount of DAHpc in 2014. Blue, hollow circles show the 10 countreis receiving the lowest amount of DAHpc in 2014.

For illustration, figure 1 shows the association between DAHpc and population size obtained from a locally weighted scatterplot smoothing regression.

### Robustness

To assess whether the results are driven by countries with very small or large population sizes, we stepwise excluded the countries with population sizes of <200 000, <500 000, <750 000, <1 million and <1.5 million, in addition to excluding the largest countries China and India. Excluding those countries (in 2014 there were 29 countries and in 1990 there were 32 countries with population sizes lower than 1.5 million) does not change the substantial interpretation of the results (see online supplementary appendix, Table A13). Furthermore, we assessed whether results were driven by countries with very small or very large population sizes by calculating influence statistics for each observation (DFBetas).^31^ DFBetas measures the difference in each parameter estimate with and without the influential point. Although online supplementary figure A2 suggests that smaller countries in tendency have a larger influence on the results, omitting country-years with a value larger than 2/sqrt(N)^31^ does not substantially change the results. As a further robustness test, we also used a set of spline variables for population size to relax the assumption of linearity. However, the spline-models confirmed that there was no significant non-linear relationship between population size and DAHpc.

The additional robustness tests, cross-sectional OLS model for the years 2010–2014, alongside the RE and FE models for the years 1990–2014, all support the results from the within-between estimator (see online supplementary appendix).

Two recent publications indicate that the size of a country—surface area in km^2^—is associated with health indices including child and maternal health.^32 33^ In one study, the effect of population size was not significant when surface area was included.^33^ However, these studies look at health coverage and inequalities as outcome variables which is affected by geography and distance (ie, remote populations could lack health services due to difficulties in effectively offering health services in areas far from regional centres), whereas our outcome variable is health aid per capita received from external funders. In our robustness check surface area was significant only for the within-country coefficient and did not change our results concerning the effect of population size on DAHpc.

We also divided the data into the different WB income groups for the year 2014 and conducted independent regressions for each group of countries. We find that the effect of population size is significant for lower and upper middle-income countries and high-income countries, but no longer for the lowest income group (LICs). This suggests that while population size plays a role in allocation of DAHpc, poverty is still a strong driver of health aid.

## Discussion

### Summary

The principal aim of this study was to assess whether and how population size of countries receiving international aid is associated with the amount of DAHpc those countries receive. Using data for 143 countries covering the years 1990–2014, we found robust evidence showing that DAHpc is substantially larger in countries with lower population size, and that increases in population size are associated with reductions in DAHpc a country receives.

### Interpretation

Our results are in line with other studies on the determinants of health aid where population size is included as a variable in the analysis.^15 16^ In a study by Fielding on the quality of governance and health aid allocation (2011), population size was negatively associated with DAHpc for 1995–2006, suggesting that population size increases of 1% are associated with reductions in DAHpc between −0.1% and −0.3%.^15^

This is in the same order of magnitude as our results for the between-country effect (−0.37). Yet, our approach took more countries into account and had a longer time series, took into account broader health estimates and used different models. Also another study focusing on allocations from the donor country South Korea,^16^ found that recipient country population size was significantly negatively related to DAH.

Our findings are also in line with at least some studies that studied total aid allocations (ie, aggregated aid to all sectors), suggesting that DAH is neither more nor less elastic than total development aid. Doucouliagos and Paldam found that total aid as percentage of GDP was negatively correlated with population size with a 1% increase in population associated with 0.48% decrease in aid allocation.^11^ Alesina and Dollar^9^ also found that small countries receive more aid per capita. Neumayer^8^ found that multilateral aid flows exhibit a bias towards less populous countries, but that the effect is non-linear in some model setups (where the strongest declines in DAH are found between small-sized and middle-sized countries).

Less populous countries may receive more DAHpc for several reasons. One reason is that funders may be more likely to allocate a fixed amount to each country, or a fixed amount relative to some other metric, such as gross national income (GNI) per capita.^34 35^ Deployment of limited resources together with a view that aid should be provided to a large number of the world’s countries,^36^ that is, each nation should at least receive something, can lead to a small population allocation bias. Moreover, donors may fear that allocating DAH in proportion to population size would make almost all DAH go to a small set of highly populous countries.

Less populous countries may also have less efficient administrations and institutions due to economies of scale and therefore need more DAHpc to provide the same health service levels or achieve the same health outcomes. Nations with smaller populations may need more funding to provide a given level of public financial management,^37^ for example, small island economies will have higher DAHpc when a minimum threshold is required for aid to be useful and efficient.^7^ Certain levels of infrastructure and staff are necessary for a national health service, that is, the costs are more or less fixed, and in larger countries these will serve a larger number of people and therefore the need for health aid per capita is lower. Also, as stated by the WB in a recent report, many small states (population size <1.5 million) lack skilled health workers, for example, in Lesotho the ratio of health workers per 1000 people is just above 1.^7^

Conversely, a certain amount of DAH may be believed to lead to more transformative change in less populous countries, meaning higher aid effectiveness. One reason therefore may be that an absolute amount of DAH represents a larger share of the resources in small countries. Also, there could be a limited capacity of large countries to absorb additional amounts of aid.^8^

Furthermore, smaller nations might potentially receive more development assistance per capita because they can be more easily held accountable for how they spend their funding due to simpler administrations and judicial, auditing and transparency frameworks—particularly when they are poor, have less tax revenues and a weaker public administration.^38^ In the case of unwanted use of funds, such as through corruption or other allocations, one may have greater difficulties penalising unwanted behaviour for larger nations, hence funders may prefer countries with smaller populations to be able to sanction unwanted country behaviour.

According to Lee and Lim,^39^ the amount of funding per health aid project has decreased over time from 1996 to 2011. Smaller projects could be easier to implement in countries with lower population size and give more visible results. As demands for measurable targets and documentation of aid effectiveness have increased over the last years, small projects in countries with lower population size might have been preferred.

Another factor is recipients’ desire to receive DAH. Populous countries may put more emphasis on independence, to be self-sufficient and thus be less likely to request or to accept support.^11^ It may be easier for a small nation state to be visible to funders and the world community at large rather than a similarly populous subnational area (eg, nations have visibility, capacities and rights that equally populated subnational regions generally do not have). In relation to that, the power distribution in international organisations might be in favour of small nations, especially in the UN General Assembly where all countries have one vote independent of size. It is well known that political and commercial influence-buying plays a role in aid allocation.^40 41^ If some donors tactically target aid to attain support in UN elections, they may focus more on smaller countries.^15^

On the funders’ side, their perceptions of many of these same factors are likely to be important. The effects of aid interventions may be higher and consequences more visible in smaller countries.^42^ Recipient countries’ visibility can direct funders to more easily visualise their efforts to others. Aid effectiveness in smaller nations might reflect higher visibility of the results rather than actual improvements in the effective use of the funding, giving the impression that the funds make more of a difference in smaller nations as opposed to larger.

Donors and other actors in global health are facing several challenges that requires a reassessment of key criteria used to allocate health aid, in a context of stagnating growth of DAH since 2010. These challenges include epidemiological change, economic transitions and the rise of MICs as well as differential growth in population size across countries.

Will a continuous ‘small-country bias’ affect health inequalities and the burden of disease globally in the years to come? An example is a recent study by the Global Burden of Disease Project estimating an index for health-related Sustainable Development Goals where India was ranked 143.^43^ India is receiving much less DAHpc (US$0.84 in 2013) than countries with a similar rank like Comoros (US$13.5 in 2013, rank 142) and Ghana (US$13.4 in 2013, rank 141). However, if DAHpc should be more evenly distributed taking into account the population size of the countries, which means more funding to MICs than LICs, alternative cofinancing obligations for the recipient countries might be considered, for example, equivalent to their share in the global economy.

The lack of transparency concerning criteria for eligibility and allocation of DAH across countries has been documented in recent studies.^44–47^ However, when allocation mechanisms are known these studies show that some funders have population size as part of their allocation formula, but others—especially bilateral funders—have not. A ‘small-country bias’ which is not openly acknowledged or discussed can contribute to the problems related to transparency and accountability. There are exceptions, however, like the focus on small states (defined as countries with population size <1.5 million) by the WB, where they explicitly argue that small states have specific challenges that need to be considered in development context.^7^ Nonetheless, no funder, to our knowledge, openly states that health is more important in small countries than in large.

Another interesting result in our analysis is the lack of correlation between the amount of DAHpc and the burden of disease (measured as DALYs), which means that there is a mismatch between the allocation of health aid and the causes of mortality and disease in the recipient country. However, this topic is beyond the scope of our study and has been addressed in other recent studies.^21 42^

Our findings point to important relations between population size and health aid per capita. This study presents theoretical and literature-based explanations for why donor countries may focus DAH transfers towards nations with smaller population size. However, our analysis does not allow one causal identification—we do not know whether low population size is the cause of greater DAHpc. Yet, the distributive aspects are clearly important, individuals in more populous country are less likely to meet their needs from health aid than those situated in less populated countries.

### Limitations

The population data from the WB and the UN are based on national censuses with 5-year or 10-year intervals, then extrapolated to cover the missing years. We have also used extrapolation methods to calculate burden of disease (DALY) annually, as these are given only in 5-year intervals. This decreases the number of independent observations in our study, which is a weakness of the analysis, particularly in the case if there should have been irregularities in the data trends which are not captured in our assessment.

Another limitation is that population health and population size might—to some degree—be jointly determined by the amount of DAH. This would result in reverse causality and thus bias the estimates for the association between population size and DAH. While this potentially could be the case, empirically the correlation between health (as measured by DALY rates in our dataset) and population size is very weak (with coefficients of −0.0549 for the correlation between DALY rates and total population size, and 0.0446 for the correlation between log DALY rates and log total population size) and insignificant. It is also important to note here that we do not claim causality in our study. The estimates should be interpreted as observed associations.

## Conclusions

No funding agency officially argues that individual need in countries with larger population sizes is less important than in countries with lower population sizes. Yet, DAHpc has been allocated disproportionately to countries with lower population sizes, revealing a potential discrepancy in the aid allocation process. In light of the new challenges and rapidly evolving landscape of DAH, global health funders are revisiting their allocation criteria. When funders reassess their criteria, they should carefully consider the role of population size and whether the small-country priority likely to be present is justifiable.
