# Supplementary material for: Do less populous countries receive more development assistance for health per capita? Longitudinal evidence for 143 countries, 1990–2014
Source: BMJ Glob Health. 2018 Jan 3;3(1):e000528. doi: 10.1136/bmjgh-2017-000528 (PMC5838402; doi:10.1136/bmjgh-2017-000528)
Supplement: Supplementary file 1 [file bmjgh-2017-000528supp001.pdf]

Supplementary appendix

Do less populous countries receive more development assistance for health per capita? Longitudinal evidence for 143 countries, 1990-2014

Lene Martinsen[1], Trygve Ottersen[1], Joseph L. Dieleman[2], Philipp Hessel[3], Jonas Minet Kinge[4], Vegard Skirbekk[4]

[1]Department of International Public Health, Norwegian Institute of Public Health, Norway
[2]Institute for Health Metrics and Evaluation (IHME), University of Washington, Seattle, USA
[3]Alberto Lleras Camargo School of Government, University of the Andes, Bogotá, Colombia
[4]Department of Epidemiology, Norwegian Institute of Public Health, Norway

Correspondence to:

Lene Martinsen: martinsen.lene@gmail.com; Vegard Skirbekk: Vegard.Skirbekk@fhi.no

Contents

Table A1: Recipient countries included in the analysis. 2

Table A2: Descriptive statistics for the variables used in the main statistical models (1990-2014) 4

Figure A1: Histograms of key variables before and after log transformation 5

Table A3: Correlation matrix for the variables included in the main statistical analysis. 8

Table A4: The collinearity measure variance inflation factor (VIF) 8

Regression without covariates (Table A5 and A6) 9

Table A7: Regression with 1 year lagged variables. 9

Robustness checks (Table A8-A11) 10

OLS, random-effects (RE) and fixed-effects (FE) regressions (Table A12) 15

Sensitivity analyses excluding countries with small and large populations (Table A13) 17

Figure A2: Influence diagnostics represented by DfBetas. 19

Table A14. Countries in aid quartiles by total health aid and by health aid per capita for the year 2014. 20

STATA commands for within-between regression 22

References 22

Table A1: Recipient countries included in the analysis.

The years each country receives DAH are specified. Income classifications: L=Low income, LM=Lower middle income, UM=Upper middle income, H=High income in 2016.

No.	Recipient country	Years with DAH	Income classification in 2016	No.	Recipient country	Years with DAH	Income classification in 2016
1	Afghanistan	1990-2014	L	73	Lesotho	1990-2014	LM
2	Albania	1992-2014	UM	74	Liberia	1990-2014	L
3	Algeria	1990-2014	UM	75	Libya	1990-2014	UM
4	Angola	1990-2014	UM	76	Lithuania	1993-96, 1998-2006	H
5	Argentina	1990-2014	UM	77	Macedonia, FYR	1992-2014	UM
6	Armenia	1990-2014	LM	78	Madagascar	1990-2014	L
7	Azerbaijan	1990-2014	UM	79	Malawi	1990-2014	L
8	Bangladesh	1990-2014	LM	80	Malaysia	1990-2014	UM
9	Barbados	1990-99, 2001,2003-2005	H	81	Maldives	1990-2014	UM
10	Belarus	1992-2014	UM	82	Mali	1990-2014	L
11	Belize	1990-2014	UM	83	Mauritania	1990-2014	LM
12	Benin	1990-2014	L	84	Mauritius	1990-2014	UM
13	Bhutan	1990-2014	LM	85	Mexico	1990-2014	UM
14	Bolivia	1990-2014	LM	86	Micronesia,	1990-95, 1997-2014	LM
15	Bosnia and	1992-2014	UM	87	Moldova	1992-2014	LM
16	Botswana	1990-2014	UM	88	Mongolia	1990-2014	LM
17	Brazil	1990-2014	UM	89	Montenegro	1992-2014	UM
18	Bulgaria	1993, 1995-2014	UM	90	Morocco	1990-2014	LM
19	Burkina Faso	1990-2014	L	91	Mozambique	1990-2014	L
20	Burundi	1990-2014	L	92	Namibia	1990-2014	UM
21	Cabo Verde	1993-95, 1997-2014	LM	93	Nepal	1990-2014	L
22	Cambodia	1990-2014	LM	94	Nicaragua	1990-2014	LM
23	Cameroon	1990-2014	LM	95	Niger	1990-2014	L
24	Central African	1990-2014	L	96	Nigeria	1990-2014	LM
25	Chad	1990-2014	L	97	Oman	1990-94, 1998-2005	H
26	Chile	1990-2014	HI	98	Pakistan	1990-2014	LM
27	China	1990-2014	UM	99	Panama	1990-2014	UM
28	Colombia	1990-2014	UM	100	Papua New	1990-2014	LM
29	Comoros	1990-2014	L	101	Paraguay	1990-2014	UM
30	Congo, Dem.	1990-2014	L	102	Peru	1990-2014	UM
31	Congo, Rep.	1990-2014	LM	103	Philippines	1990-2014	LM
32	Costa Rica	1990-2014	UM	104	Poland	1992-2002, 2006-07	H
33	Cote d'Ivoire	1990-2014	LM	105	Romania	1992-2014	UM
34	Croatia	1994-2007	HI	106	Russian	1992-2011	UM
35	Cuba	1990-2014	UM	107	Rwanda	1990-2014	L
36	Czech Republic	1992-93, 1995, 1998-	HI	108	Samoa	1990-2014	LM
37	Djibouti	1990-2014	LM	109	Sao Tome and Principe	1990-2014	LM
38	Dominican	1990-2014	UM	110	Saudi Arabia	1998-2003	H
39	Ecuador	1990-2014	UM	111	Senegal	1990-2014	L
40	Egypt, Arab	1990-2014	LM	112	Serbia	1992-2014	UM
41	El Salvador	1990-2014	LM	113	Sierra Leone	1990-2014	L
42	Equatorial Guinea	1990-2014	UM	114	Slovak Republic	1993, 1998, 2004-2006	H
43	Eritrea	1990-2014	L	115	Solomon Islands	1990-2014	LM
44	Estonia	1996-2000, 2003-05	HI	116	South Africa	1990-2014	UM

45	Ethiopia	1990-2014	L	117	Sri Lanka	1990-2014	LM
46	Fiji	1990-2014	UM	118	St. Lucia	1990-2014	UM
47	Gabon	1990-2014	UM	119	St. Vincent and the Grenadines	1990-2014	UM
48	Gambia, The	1990-2014	L	120	Sudan	1990-2014	LM
49	Georgia	1990-2014	UM	121	Suriname	1990-2014	UM
50	Ghana	1990-2014	LM	122	Swaziland	1990-2014	LM
51	Grenada*	1990-2014	UM	123	Syrian Arab	1990-2014	LM
52	Guatemala	1990-2014	LM	124	Tajikistan	1990-2014	LM
53	Guinea	1990-2014	L	125	Tanzania	1990-2014	L
54	Guinea-Bissau	1990-2014	L	126	Thailand	1990-2014	UM
55	Guyana	1990-2014	UM	127	Timor-Leste	1990-2014	LM
56	Haiti	1990-2014	L	128	Togo	1990-2014	L
57	Honduras	1990-2014	LM	129	Tonga	1990-2014	LM
58	Hungary	1993-2001, 2012	H	130	Trinidad and Tobago	1990-2005	H
59	India	1990-2014	LM	131	Tunisia	1990-2014	LM
60	Indonesia	1990-2014	LM	132	Turkey	1990-2014	UM
61	Iran, Islamic	1990-2014	UM	133	Turkmenistan	1990-2014	UM
62	Iraq	1993-2014	UM	134	Uganda	1990-2014	L
63	Jamaica	1990-2014	UM	135	Ukraine	1992-2014	LM
64	Jordan	1990-2014	UM	136	Uruguay	1990-2014	H
65	Kazakhstan	1990-2014	UM	137	Uzbekistan	1990-2014	LM
66	Kenya	1990-2014	LM	138	Vanuatu	1990-2014	LM
67	Kiribati	1990-2014	LM	139	Venezuela, RB	1990-2014	UM
68	Korea, Rep.	1990, 1992-94, 1998-2000	H	140	Vietnam	1990-2014	LM
69	Kyrgyz Rep.	1990-2014	LM	141	Yemen, Rep.	1990-2014	LM
70	Lao PDR	1990-2014	LM	142	Zambia	1990-2014	LM
71	Latvia	1993-96, 1998-2004, 2010-11	H	143	Zimbabwe	1990-2014	L
72	Lebanon	1990-2014	UM				

A number of adjustments was done to the original dataset downloaded from IHME due to missing data: 1) Bahrain, Malta and Slovenia were dropped because of missing DAH data (Bahrain received DAH only 1998-2000, Malta received DAH only in 1994, Slovenia is listed with zero DAH in the IHME database). 2) The following countries were excluded from the pooled OLS regression (average 2010-2014) because of transition from low- and middle-income to high-income country from 2009 (according to Dieleman et al. 2014): Barbados, Croatia, Czech Republic, Equatorial Guinea, Estonia, Hungry, Latvia, Oman, Poland, Republic of Korea, Saudi Arabia, Slovak Rep, and Trinidad & Tobago. 3) Some countries/areas were deleted from the DAH dataset because no data was available for any of the explanatory variables: Anguilla, Cook Island, French Guiana, Mayotte, Nauru, Netherlands Antilles, Niue, Northern Mariana Islands, St. Helena, Tokelau, Turks and Caicos Islands, and Wallis & Futuna. Most of these countries are overseas territories of previous colonial powers. 4) The following countries were removed from the analysis because they missed one or more of the covariates for five data points or more (some for the entire time period 1990-2014): Antigua & Barbuda, Dominica, Dem. People's of Korea, Kosovo, Marshall Island, Myanmar, Palau, Seychelles, Somalia, St Kitts & Nevis, South Sudan, Tuvalu, and West Bank & Gaza.

Table A2: Descriptive statistics for the variables used in the main statistical models (1990-2014)

The table show both the actual values and the natural logs. NB: Eritrea lacks estimates of population size for 2012-2014 reducing the number of observations of DAHpc in the analyses from 3,575 to 3,572.

Variable	Description	# of obs	Mean	Standard deviation	Minimum	Maximum
**DAH**	Development assistance for health, total (USD)	3,575	US$ 72 million	US$152 million	US$0	US$1.570 million
**DAHpc**	Development assistance for health, per capita (USD)	3,572	US$ 7.70	US$ 18.74	US$ 0	US$ 379.55
**logDAHpc**		3,572	0.77	1.99	-9.59	5.94
**POP**	Population size	3,572	36.8 million	141 million	72.4k	1.36 billion
**logPOP**		3,572	15.7	1.9	11.19	21.03
**GDPpc**	Gross domestic product per capita (USD)	3,572	3472.4	3780.3	86.7	24446.5
**logGDPpc**		3,572	7.6	1.1	4.5	10.1
**DALYR**	Rate of disability-adjusted life years, age-standardized	3,575	48264.66	26471.02	17129.33	187343.7
**logDALYR**		3,575	0.49	1.14	9,7	12.14
**U5MR**	Under 5 mortality rate	3,575	65.15	56.9	3.2	328.2
**logU5MR**		3,575	3.77	0.96	1.16	5.79
**MMR**	Maternal mortality rate	3,575	304.28	372.0	3	2900
**log(MMR**		3,575	4.9	1.4	1.1	8.0
**DTP3**	Immunization coverage of DTP3	3,572	0.77	0.21	0.05	1.0
**TFR**	Total fertility rate	3,575	3.8	1.8	1.1	8.8

Figure A1: Histograms of key variables before and after log transformation

Left figures: Before log transformation. Right figures: After log transformation.

A. Development Assistance for Health per capita (DAHpc)

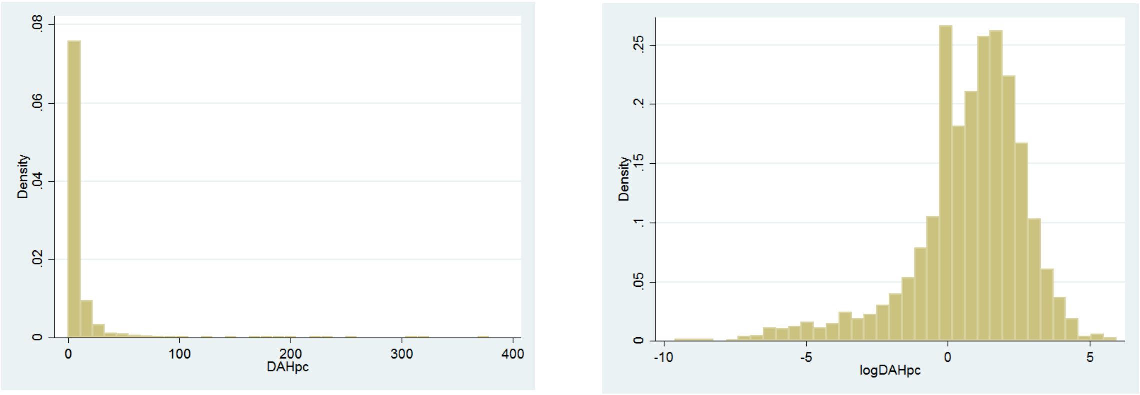

B. Population size

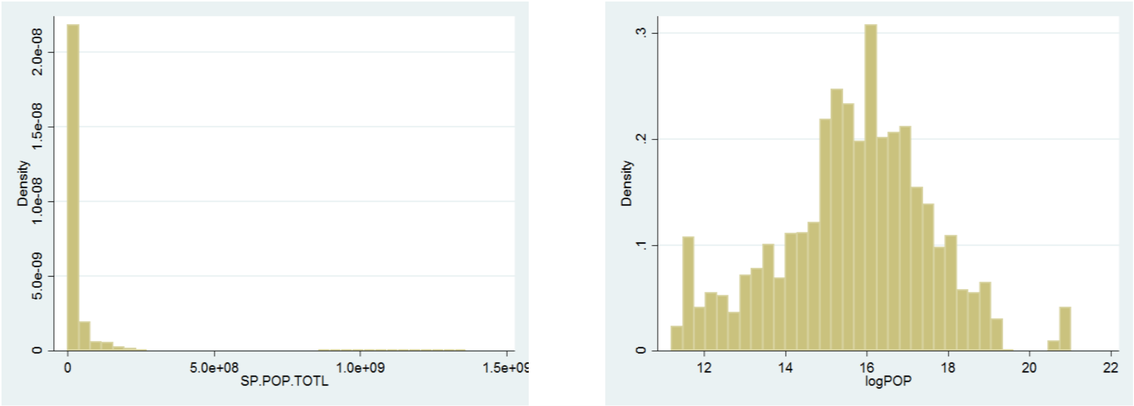

C. Gross Domestic Product per capita (GDPpc)

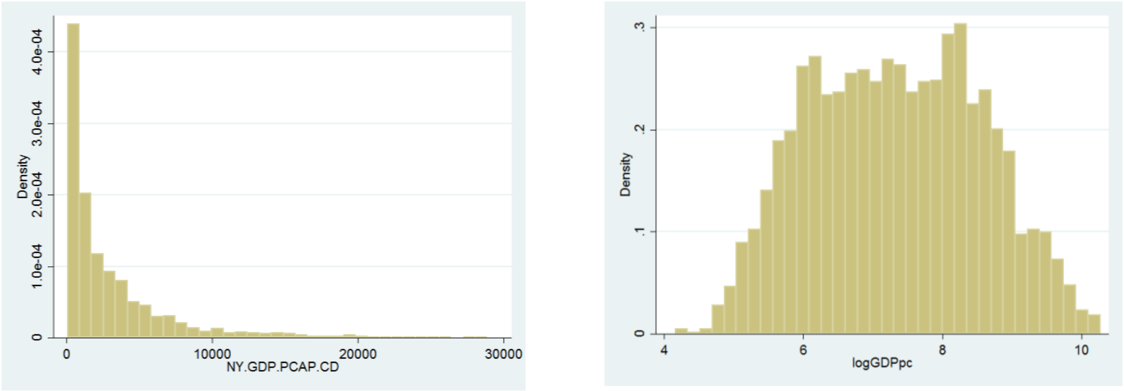

D. Disability-Adjusted Life Years Rate (DALYR)

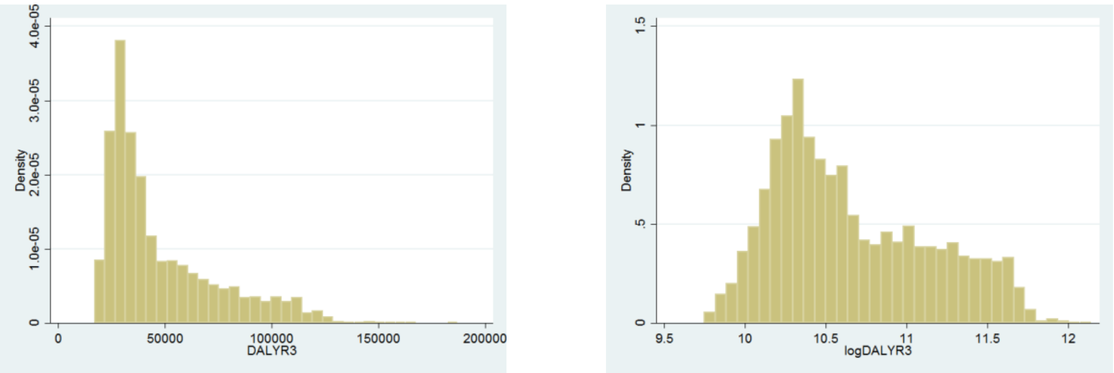

E. Under 5 Mortality Rate (U5MR)

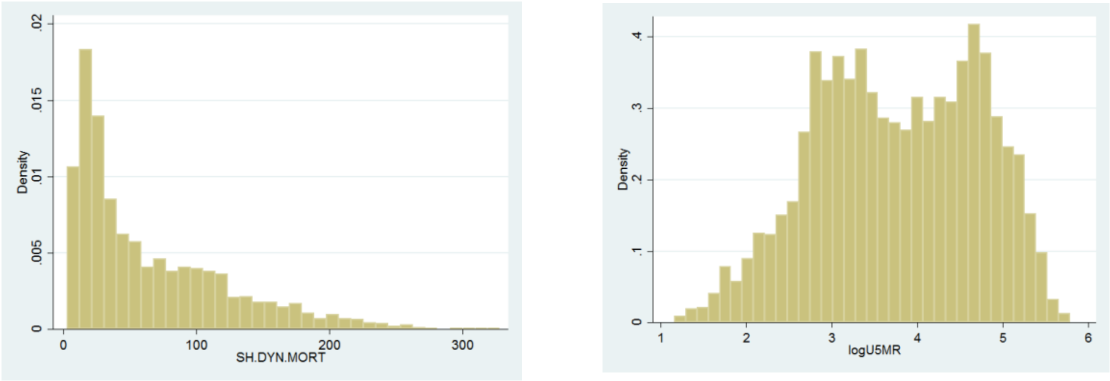

F. Maternal Mortality Rate (MMR)

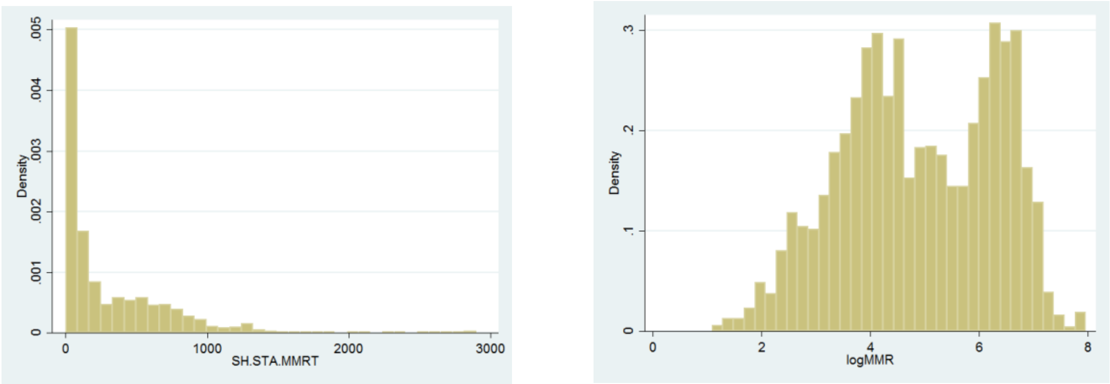

E. Immunization coverage of DTP3

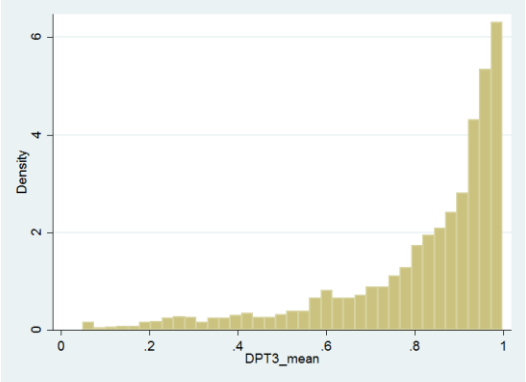

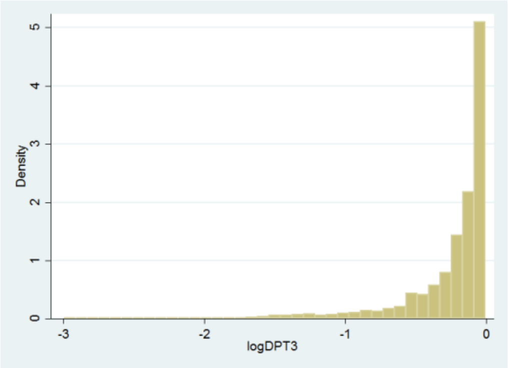

F. Fertility

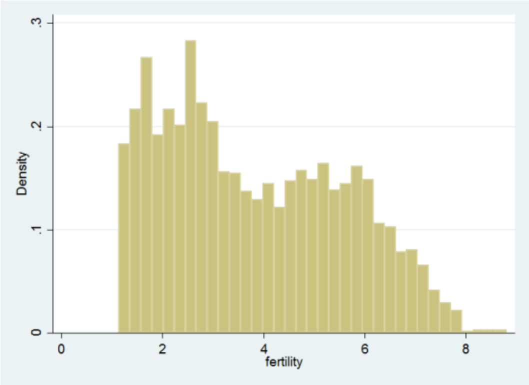

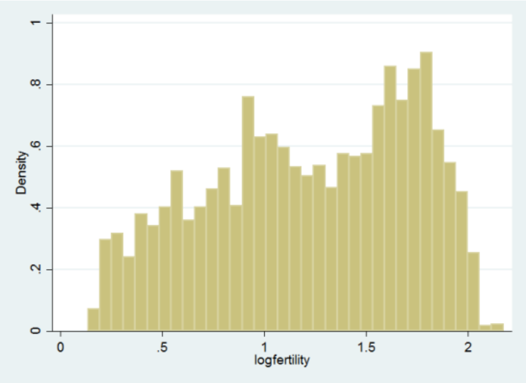

Table A3: Correlation matrix for the variables included in the main statistical analysis.

Based on the complete data set for 1990-2014. Log transformed variables (except fertility and DTP3)

	logDAHpc	logPOP	logGDP~D	logDALYR	logU5MR	logMMR	DTP3	fertility
logDAHpc	1.00							
logPOP	-0.28	1.00						
logGDPpcUSD	-0.27	-0.11	1.00					
logDALYR	0.22	0.04	-0.70	1.00				
logU5MR	0.25	0.07	-0.80	0.83	1.00			
logMMR	0.35	0.04	-0.78	0.78	0.91	1.00		
DTP3	-0.09	-0.02	0.51	-0.64	-0.67	-0.64	1.00	
fertility	0.26	-0.04	-0.73	0.75	0.85	0.84	-0.65	1.00

Table A4: The collinearity measure variance inflation factor (VIF).

A. For the covariates included in the main regression analysis. B. Including age structure of the population (PopAge0_14)

**A.**			**B.**		
**Variable**	**VIF**	**1/VIF**	**Variable**	**VIF**	**1/VIF**
logPOP	1.06	0.94	*logPOP*	1.12	0.89
logGDPpcUSD	3.05	0.33	*logGDPpcUSD*	3.06	0.33
logDALYR3	3.44	0.29	*logDALYR3*	5.84	0.17
logU5MR	8.89	0.11	*logU5MR*	11.43	0.09
logMMR	6.83	0.15	*logMMR*	7.43	0.13
DTP3_mean	1.99	0.50	*DTP3_mean*	2.10	0.48
fertility	4.48	0.22	*fertility*	14.44	0.07
			PopAge0_14	17.14	0.06

Regression without covariates (Table A5 and A6)

The within-between regression was conducted without the covariates.

Table A5. Regression without covariates including a lagged dependent variable (L1.logDAHpc)

	Coef.	P-value	CI
L1.logDAHpc	0.76	0.00	0.72, 0.80
logPOP_demean	-0.59	0.02	-1.09, -0.09
logPOP_mean	-0.08	0.00	-0.11, -0.05
Number of obs = 3,429			
Number of groups = 143			

Table A6. Regression without covariates and without a lagged dependent var (i.e. not inclu L1.logDAHpc)

	Coef.	P-value	CI
logPOP_demean	-1.90	0.01	-3.34, -0.45
logPOP_mean	-0.31	0.00	-0.42, -0.21
Number of obs = 3,429			
Number of groups = 143			

Table A7: Regression with 1 year lagged variables.

Since the allocation of development assistance can be affected by what the recipient countries received the previous year(s) we did a regression with a 1 year lagged dependent variable (L1.logDAHpc) included as a covariate. We also lagged the other covariates in the same regression.

When a lagged dependent variable is included in the regression (L1.logDAHpc) both the within-country and the between-country are negatively significant, however, the size of the coefficients have decreased as compared to the main regression without the lagged dependent variable.

Table A7. Within-between regressions where the covariates are lagged with 1 year. The outcome variable is average log DAHpc. Column A includes a 1 year lagged dependent variable (L1.logDAHpc).

	A. Incl. 1 year lagged dependent var		B. Without 1 year lagged dependent var	
Covariates	**Coef.**	**P>|z|**	**Coef.**	**P>|z|**
L1.logDAHpc	**0.68**	0.00		
logPOP_mean	**-0.12**	0.00	**-0.35**	0.00
logPOP_demean	**-0.84**	0.01	**-2.71**	0.00
logGDPpcUSD_mean	**-0.09**	0.02	**-0.21**	0.07
logGDPpcUSD_demean	**-0.37**	0.00	**-0.57**	0.01
logDALYR3_mean	-0.11	0.34	-0.23	0.52
logDALYR3_demean	0.14	0.58	0.03	0.97
logU5MR_mean	0.09	0.29	0.07	0.79
logU5MR_demean	0.23	0.27	0.46	0.36
logMMR_mean	**0.19**	0.00	**0.70**	0.00
logMMR_demean	**-0.30**	0.03	**-0.65**	0.08
DTP3_mean2	0.24	0.20	**0.96**	0.08
DTP3_demean	0.44	0.11	**1.78**	0.04
fertility_mean	-0.05	0.13	-0.10	0.29
fertility_demean	0.00	0.94	-0.01	0.95

Robustness checks (Table A8-A11)

We conducted several robustness checks to test a number of covariates that were not part of the main regression analysis. The regression is the same as in the main analysis using the within-between estimator (see main text page 10).

Table A8: Description of covariates tested in robustness checks. HIVprev and GHEgdp were log transformed (natural log) because of their right-skewed distribution.

Variable name	Description	Source
**Log HIVprev**	Natural log of prevalence of HIV, total (% of population ages 15-49)	WB
**Water**	Improved water source (% of population with access)	WB
**VA**	Voice and accountability captures perceptions of the extent to which a country's citizens are able to participate in selecting their government, as well as freedom of expression, freedom of association, and a free media	World Wide Governance (wgi) indicator
**PSAV**	Political Stability and Absence of Violence/Terrorism measures perceptions of the likelihood of political instability and/or politically motivated violence, including terrorism	World Wide Governance (wgi) indicator
**Log GHEgdp**	Health expenditure, public/government (% of GDP)	WB
**LE**	Life expectancy at birth, total (years)	WB
**Surface area**	A country's total area, including areas under inland bodies of water and some coastal waterways.	WB

Table A9: Regression results from robustness checks with additional covariates. The coefficients in bold are significant at $p < 0.05$.

Covariates	**HIV**		**VA**		**PSAV**		**Water**		**GHEgdp**		**LE**		**Area**		**All**	
	**Coef.**	**z**	**Coef.**	**z**	**Coef.**	**z**	**Coef.**	**z**	**Coef.**	**z**	**Coef.**	**z**	**Coef.**	**z**	**Coef.**	**z**
logPOP_mean	**-0.37**	-7.63	**-0.33**	-7.36	**-0.39**	-7.44	**-0.37**	-8.58	**-0.32**	-6.87	**-0.37**	-8.41	**-0.35**	-7.59	**-0.37**	-6.89
logPOP_demean	**-2.49**	-2.71	-1.55	-1.38	-0.91	-0.81	**-2.16**	-2.51	**-1.84**	-1.86	**-2.30**	-2.85	**-2.38**	-2.97	**-2.79**	-2.37
logGDPpcUSD_mean	**-0.34**	-2.74	**-0.52**	-4.48	**-0.46**	-3.77	**-0.30**	-2.54	**-0.42**	-3.72	**-0.27**	-2.14	**-0.25**	-2.19	**-0.38**	-3.13
logGDPpcUSD_demean	-0.12	-0.51	-0.25	-0.72	-0.17	-0.48	-0.14	-0.49	-0.17	-0.53	-0.24	-1.15	-0.19	-0.96	-0.01	-0.01
logDALYR3_mean	**-1.39**	-3.41	-0.43	-1.39	-0.28	-0.78	-0.20	-0.52	-0.54	-1.47	0.15	0.25	-0.22	-0.59	-0.59	-1.11
logDALYR3_demean	0.07	0.09	0.11	0.17	0.42	0.71	0.03	0.05	-0.26	-0.40	0.11	0.19	-0.13	-0.21	0.28	0.49
logU5MR_mean	**0.67**	1.86	**0.60**	2.58	0.34	1.18	0.12	0.46	**0.50**	1.95	0.11	0.40	0.10	0.36	**1.26**	4.02
logU5MR_demean	-0.24	-0.44	0.14	0.32	-0.20	-0.50	0.28	0.60	0.24	0.58	**0.77**	2.04	0.57	1.24	0.22	0.48
logMMR_mean	0.14	0.70	**0.30**	1.86	**0.42**	2.32	**0.61**	3.43	**0.43**	2.56	**0.63**	3.22	**0.63**	3.48	-0.11	-0.61
logMMR_demean	-0.46	-0.89	-0.63	-1.58	-0.58	-1.51	-0.37	-0.85	-0.50	-1.26	-0.66	-1.63	-0.59	-1.45	-0.48	-0.86
DTP3_mean2	0.01	1.12	0.00	0.54	0.00	0.39	0.01	1.18	0.00	-0.38	0.01	1.39	0.01	1.36	0.01	1.10
DTP3_demean	**0.01**	2.05	0.00	0.54	0.00	0.74	0.01	1.66	0.00	0.46	0.01	1.53	0.01	1.79	0.00	0.85
fertility_mean	0.02	0.25	-0.10	-0.97	-0.16	-1.40	-0.09	-0.94	-0.14	-1.28	-0.06	-0.59	-0.08	-0.83	-0.06	-0.70
fertility_demean	-0.11	-0.76	-0.18	-1.02	-0.12	-0.73	-0.08	-0.57	-0.08	-0.56	-0.14	-1.04	-0.10	-0.79	-0.12	-0.56
logHIVprev_mean	**0.30**	4.33													**0.28**	4.67
logHIVprev_demean	-0.06	-0.49													-0.12	-0.82
VA_est_mean			**0.54**	5.01											**0.42**	3.62
VA_est_demean			0.24	1.27											0.09	0.43
PSAV_est_mean					0.13	0.95									-0.26	-1.79
PSAV_est_demean					0.14	1.53									0.09	0.81
water_mean							0.00	0.31							-0.01	-1.45
water_demean							0.01	0.52							0.03	1.65
logGHEgdp_mean									**0.66**	3.74					**0.51**	2.34
logGHEgdp_demean									0.19	1.38					0.10	0.69

LE_mean											0.03	0.70			0.04	1.17
LE_demean											0.03	1.29			**0.08**	2.23
Area_mean													0.00	-1.18	0.00	-0.17
Area_demean													**0.00**	-2.66	**0.00**	-3.04

Table A10: Regression results from robustness checks with the data set divided into the income categories of the World Bank as categorised in 2014. LICs = Low-income countries, LMICs = Low middle-income countries, UMICs = Upper middle-income countries, HICs = High-income countries. The coefficients in bold are significant at $p < 0.05$.

	LICs		*LMICs*		*UMICs*		*HICs*	
Covariates	Coef.	P-value	Coef.	P-value	Coef.	P-value	Coef.	P-value
logPOP_mean	-0.03	0.65	**-0.41**	0.00	**-0.35**	0.00	**-0.32**	0.00
logPOP_demean	1.52	0.32	**-5.63**	0.00	**-4.40**	0.00	-2.13	0.56
logGDPpcUSD_mean	0.23	0.21	-0.27	0.28	-0.29	0.46	-0.98	0.19
logGDPpcUSD_demean	-0.16	0.70	-0.88	0.11	-0.07	0.89	0.00	1.00
logDALYR3_mean	0.57	0.41	-0.61	0.41	0.36	0.67	-0.97	0.07
logDALYR3_demean	0.36	0.31	-0.62	0.58	0.54	0.69	0.05	0.99
logU5MR_mean	0.45	0.45	-0.10	0.84	-0.53	0.39	0.14	0.81
logU5MR_demean	0.27	0.59	0.52	0.48	0.27	0.71	0.90	0.57
logMMR_mean	-0.22	0.45	**0.83**	0.00	**0.97**	0.00	0.59	0.20
logMMR_demean	**-1.30**	0.00	**-1.06**	0.03	-0.48	0.36	-0.75	0.32
DTP3_mean2	**1.71**	0.00	**1.70**	0.03	-1.55	0.44	0.68	0.78
DTP3_demean	1.38	0.13	1.25	0.43	2.42	0.13	7.62	0.12
fertility_mean	-0.10	0.44	0.01	0.94	-0.19	0.43	**-0.38**	0.03
fertility_demean	-0.17	0.36	0.33	0.23	-0.08	0.74	-0.22	0.60

Table A11. Regression results from robustness checks excluding under 5 mortality rate (U5MR) and maternal mortality rate (MMR). The coefficients in bold are significant at $p < 0.05$.

	Coef.	*P-value*
logPOP_mean	**-0.34**	0.00
logPOP_demean	**-2.63**	0.00
logGDPpcUSD_mean	**-0.42**	0.00
logGDPpcUSD_demean	-0.20	0.34
logDALYR3_mean	0.27	0.43
logDALYR3_demean	-0.32	0.53
DTP3_mean2	0.53	0.35
DTP3_demean	**1.94**	0.03
fertility_mean	0.19	0.07
fertility_demean	-0.05	0.76
Number of obs.	3,572	
Number of countries	143	

Table A12. Regression result from robustness check excluding the countries that were categorized as high-income countries (HICs) by the World Bank in 2016 (see Table A1 in Supp. App. for details on which countries are HICs). The coefficients in bold are significant at $p < 0.05$.

	Coef.	*P-value*
logPOP_mean	**-0.35**	0.00
logPOP_demean	**-3.95**	0.00
logGDPpcUSD_mean	-0.16	0.21
logGDPpcUSD_demean	-0.33	0.09
logDALYR3_mean	-0.10	0.83
logDALYR3_demean	-0.51	0.38
logU5MR_mean	-0.04	0.90
logU5MR_demean	0.36	0.38
logMMR_mean	**0.69**	0.00
logMMR_demean	**-0.73**	0.04
DTP3_mean2	**1.22**	0.03
DTP3_demean	**1.62**	0.05
fertility_mean	-0.03	0.83
fertility_demean	0.07	0.65
Number of obs.	3,222	
Number of countries	129	

OLS, random-effects (RE) and fixed-effects (FE) regressions (Table A12)

For the cross-sectional analyses we first averaged information on all variables over the years 2010-2014 and then used an Ordinary Least Squares (OLS) model to regress health-related development assistance per capita (DAHpc) on population size and covariates. The model can be written as:

$$logDAHpc_i=\alpha+ \beta_1 logPOP_i+\beta_2\boldsymbol{X}_i +\varepsilon \quad (1)$$

Whereas $logDAHpc_i$ is the log of health-related development assistance per capita in country *i*, $logPOP_i$ is log of the population size, $\boldsymbol{X}_i$ is a vector of covariates, α is the intercept and ε_i the error term.

For the longitudinal analyses we used data for the period 1990-2014.

The random-effects model can be written as:

$$logDAHpc_{it}=\alpha+\beta_1 logPOP_{it-1}+ \beta_2\boldsymbol{X}_{it-1} +\beta_3 Year_t + \varepsilon_{it} + \mu_i \qquad (2)$$

Whereas $logDAHpc_{it}$ is the log of health-related development assistance per capita in country *i* in year *t*, $logPOP_{it-1}$ is log of the population size of country *i* in the preceding year

(t-1), $\boldsymbol{X}_i$ is a vector of covariates, $Year_t$ is a dummy for calendar year, α is the intercept, ε_{it} the within-country error term and μ_{it} the between-country error term.

The fixed-effects model can be written as:

$$logDAHpc_{it}=\alpha_i+\beta_1 logPOP_{it-1}+\beta_2\boldsymbol{X}_{it-1}+\beta_3 YEAR_t+\varepsilon_{it} \quad (3)$$

Whereas the only difference to the random-effects model shown in equation 2 is that α_i is a country-specific fixed-effect, capturing all unobserved differences between countries. (ε_{it}) is the general error.

Country-clustered robust standard errors were used in all the regression models.

The vector of covariates is given by

$$\boldsymbol{X}_i = \beta_3 logGDPpc_i+\beta_5 logDALYR_i+\beta_6 logU5MR_i\, etc+\beta_7 logMMR_i+\beta_8 DTP3_i+\beta_9 TFR_i$$

Table A12. Summary of the regressions conducted for robustness checks. The outcome variable is average log DAHpc. The results do not change substantially if we lag the variables with 1 year. The coefficients in bold are significant at $p < 0.05$.

	**Fixed effects**		**Random effects**		**OLS**	
Covariates	Coef.	P>|t|	Coef.	P>|z|	Coef.	P>|t|
logPOP	**-2.62**	0.00	**-0.37**	0.00	**-0.38**	0.00
logGDPpcUSD	-0.31	0.14	**-0.28**	0.06	**-0.54**	0.00
logDALYR3	-0.24	0.71	0.20	0.57	0.59	0.09
logU5MR	0.34	0.45	0.05	0.88	1.04	0.20
logMMR	-0.50	0.19	0.08	0.68	0.31	0.27
DTP3_mean	**1.94**	0.04	0.87	0.25	0.10	0.54
fertility	-0.02	0.89	0.17	0.16	-0.10	0.43
	3547		3,547		143	
	143		143		143	

The results from the cross-sectional pooled OLS (Ordinary Least Squares) show a significant association between the average amount of DAHpc received over the last 5 years (2010-2014) and average population size for 2010-2014. This analysis gives a snapshot of the situation averaged over 5 years, instead of looking at the change over time.

Figure A1: Scatter plot of the natural log of DAHpc and natural log of population size.

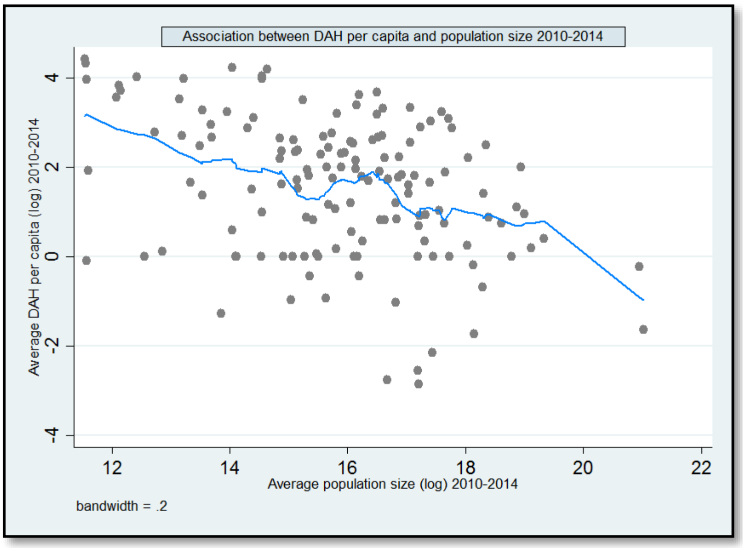

The results of the RE and FE models, covering the years 1990-2014, show that increases in population size over time are significantly associated with decreases in DAHpc supporting the results from the within-between estimator. The RE model suggests that a one percent increase in population size is associated with about 0.4 percent decrease in DAHpc (-0.41, 95% CI=-0.50, -0.31). In comparison, the FE model shows that a one percent increase in population size was associated with a 2.1% decrease in DAHpc (-2.06, 95% CI=-3.62, -0.50).

Sensitivity analyses excluding countries with small and large populations (Table A13)

To assess whether the results are driven by countries with very small or large population sizes, we stepwise excluded the countries with population sizes of less than 200,000, less than 500,000, less than 750,000, less than one million, and less than 1.5 million, in addition to excluding the largest countries China and India.

The association between logDAHpc and logPOP stays significant and negative for both the between-country variation and the within-country variation of population size for all the sensitivity analyses.

.

Table A13. Sensitivity analysis excluding countries with small population sizes and the two largest countries India and China. Five different regressions were conducted with the exclusion criteria defined above each column. The coefficients in bold are significant at $p < 0.05$.

Exclusion criteria:	*1. Pop smaller than 200,000 & India & China*		*2. Pop smaller than 500,000 & India & China*		*3. Pop smaller than 750,000 & India & China*		*4. Pop smaller than 1 million & India & China*		*5. Pop smaller than 1.5 million & India & China*	
Covariates	Coef.	P>|z|	Coef.	P>|z|	Coef.	P>|z|	Coef.	P>|z|	Coef.	P>|z|
logPOP_mean	**-0.36**	0.00	**-0.36**	0.00	**-0.31**	0.00	**-0.32**	0.00	**-0.37**	0.00
logPOP_demean	**-1.82**	0.04	**-2.10**	0.03	**-2.00**	0.05	**-2.01**	0.06	**-1.97**	0.07
logGDPpcUSD_mean	**-0.26**	0.02	**-0.24**	0.04	**-0.23**	0.06	**-0.22**	0.07	-0.15	0.22
logGDPpcUSD_demean	-0.12	0.59	-0.16	0.56	-0.12	0.69	-0.10	0.76	0.00	1.00
logDALYR3_mean	-0.14	0.73	-0.21	0.59	-0.19	0.63	-0.24	0.54	-0.22	0.59
logDALYR3_demean	0.29	0.65	0.18	0.79	0.18	0.80	0.15	0.84	0.09	0.90
logU5MR_mean	0.09	0.76	0.12	0.69	0.15	0.61	0.17	0.57	0.19	0.54
logU5MR_demean	0.08	0.88	0.13	0.82	0.19	0.74	0.19	0.76	0.19	0.76
logMMR_mean	**0.63**	0.00	**0.67**	0.00	**0.69**	0.00	**0.72**	0.00	**0.77**	0.00
logMMR_demean	-0.52	0.23	-0.45	0.34	-0.46	0.34	-0.46	0.34	-0.46	0.36
DTP3_mean2	**0.01**	0.17	**0.01**	0.06	**0.01**	0.04	**0.01**	0.04	**0.01**	0.08
DTP3_demean	**0.01**	0.04	**0.01**	0.04	**0.01**	0.06	**0.01**	0.05	**0.01**	0.08
fertility_mean	-0.12	0.26	-0.11	0.30	-0.13	0.22	-0.14	0.18	-0.17	0.12
fertility_demean	-0.12	0.39	-0.07	0.63	-0.06	0.68	-0.07	0.62	-0.05	0.77
Number of obs	3,207		3,059		2,955		2,879		2,722	
Number of countries	133		129		125		120		114	

Figure A2: Influence diagnostics represented by DfBetas.

The graph shows the DfBetas of logPOP (Y axis) against population size in millions (X axis). The cut-off value (solid red line) is set at 2/sqrt(N) (Belsley, Kuh, & Welsch, 2005).

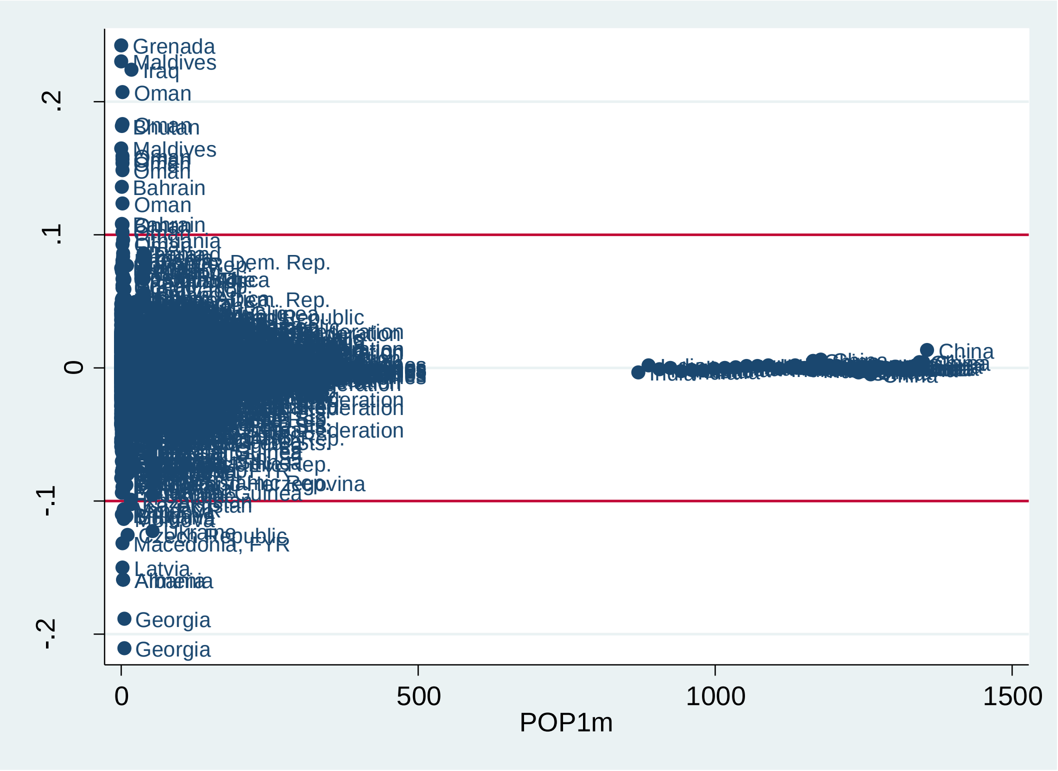

Table A14. Countries in aid quartiles by total health aid and by health aid per capita for the year 2014.

Quantile 1				Quantile 2			
Total DAH		DAHpc		Total DAH		DAHpc	
0-5.6M		0-1		5.6M-3.74B		1-5.9	
Country	USD	Country	USD	Country	USD	Country	USD
Barbados	0	Barbados	0	Algeria	5742057	Turkmenistan	1.020069
Croatia	0	Croatia	0	Tunisia	5835220	Grenada	1.041503
Czech Republic	0	Czech Republic	0	Comoros	6481960	Indonesia	1.090963
Estonia	0	Estonia	0	Serbia	6861301	Macedonia, FYR	1.109494
Hungary	0	Hungary	0	Bulgaria	7193597	Peru	1.330916
Korea, Rep.	0	Korea, Rep.	0	Cuba	9483863	Iraq	1.33726
Latvia	0	Latvia	0	Sao Tome and Principe	9680627	Belarus	1.401348
Lithuania	0	Lithuania	0	Gabon	1.00E+07	Thailand	1.534564
Oman	0	Oman	0	Syrian Arab Republic	1.04E+07	Kazakhstan	1.579174
Poland	0	Poland	0	Djibouti	1.17E+07	Mauritius	1.734115
Russian Federation	0	Russian Federation	0	Congo, Rep.	1.22E+07	Morocco	1.883379
Saudi Arabia	0	Saudi Arabia	0	Guyana	1.22E+07	Paraguay	1.981921
Slovak Republic	0	Slovak Republic	0	Iran, Islamic Rep.	1.28E+07	Pakistan	2.095333
Trinidad and Tobago	0	Trinidad and Tobago	0	Solomon Islands	1.29E+07	Uzbekistan	2.109725
Maldives	52051.23	Venezuela, RB	0.0177404	Paraguay	1.30E+07	Azerbaijan	2.199615
Grenada	110774.3	Chile	0.0335347	Belarus	1.33E+07	Philippines	2.271546
Uruguay	321218.2	Libya	0.0845187	Albania	1.46E+07	Ukraine	2.378618
Equatorial Guinea	358973.8	Uruguay	0.0939359	Vanuatu	1.48E+07	Congo, Rep.	2.511647
Libya	524363.3	Argentina	0.0985872	Fiji	1.63E+07	Sri Lanka	2.574262
Venezuela, RB	545311.7	China	0.1017637	Cabo Verde	1.69E+07	Ecuador	2.620834
Chile	590674.2	Maldives	0.1298036	Jamaica	1.70E+07	Montenegro	2.989629
Costa Rica	1342078	Malaysia	0.143647	Armenia	1.96E+07	Vietnam	3.134569
Montenegro	1858981	Algeria	0.1468057	Azerbaijan	2.10E+07	Bangladesh	3.330098
Mauritius	2186605	Iran, Islamic Rep.	0.1631076	Guinea-Bissau	2.16E+07	Sudan	3.551179
St. Vincent & the G.	2294787	Costa Rica	0.2820928	Mauritania	2.45E+07	Bhutan	3.752283
Macedonia, FYR	2304968	Equatorial Guinea	0.3178379	Panama	2.54E+07	El Salvador	4.233969
Kiribati	2813291	Turkey	0.3402439	Turkey	2.62E+07	Angola	4.33567
Belize	2820189	Egypt, Arab Rep.	0.3755396	El Salvador	2.66E+07	Bolivia	4.838048
Bhutan	2913453	Tunisia	0.5236242	Timor-Leste	2.70E+07	Albania	5.040361
Samoa	3160643	Syrian Arab Republic	0.5419289	Kazakhstan	2.73E+07	Gabon	5.357855
Tonga	3615596	India	0.5519675	Georgia	3.13E+07	Guatemala	5.403879
Suriname	3688366	Mexico	0.6402915	Mongolia	3.16E+07	Lebanon	5.788247
St. Lucia	3838750	Cuba	0.8290259	Lebanon	3.24E+07	Cameroon	5.871688
Argentina	4237425	Brazil	0.9533336	Egypt, Arab Rep.	3.45E+07	Madagascar	5.87254
Malaysia	4342164	Serbia	0.9622365	Bosnia and Herzegovina	3.47E+07	Chad	5.927221
Turkmenistan	5575944	Bulgaria	0.9958	Gambia, The	3.74E+07	Jamaica	5.956088

Quantile 3				Quantile 4			
Total DAH		DAHpc		Total DAH		DAHpc	
3.75B-14.2B		5.9-15.27		14.2B-136B		15.27-380	
Country	USD	Country	USD	Country	USD	Country	USD
Micronesia, Fed. Sts.	3.95E+07	Togo	5.975571	Burkina Faso	1.55E+08	Guyana	16.00967
Eritrea	4.11E+07	Mauritania	6.033739	Romania	1.55E+08	Samoa	16.43686
Peru	4.12E+07	Yemen, Rep.	6.241626	Yemen, Rep.	1.64E+08	South Africa	16.48003
Ecuador	4.17E+07	Panama	6.506588	Papua New Guinea	1.69E+08	Afghanistan	16.48705
Togo	4.32E+07	Suriname	6.731479	Brazil	1.95E+08	Guinea	16.94628
Iraq	4.68E+07	Armenia	6.733867	Guinea	2.00E+08	Colombia	17.2641
Central African Republic	4.77E+07	Nepal	7.20774	Cambodia	2.02E+08	Fiji	18.34728
Bolivia	5.11E+07	Tajikistan	7.272633	Nepal	2.04E+08	Gambia, The	19.5017
Sri Lanka	5.35E+07	Niger	7.405478	Sierra Leone	2.10E+08	St. Vincent and G.	20.98437
Moldova	5.43E+07	Nigeria	7.702089	Liberia	2.22E+08	Uganda	21.23071
Kyrgyz Republic	5.92E+07	Romania	7.787076	Philippines	2.27E+08	Papua New Guinea	21.72769
Tajikistan	6.08E+07	Belize	8.018871	Senegal	2.27E+08	St. Lucia	21.75903
Morocco	6.46E+07	Georgia	8.393445	Mali	2.44E+08	Tanzania	22.17037
Uzbekistan	6.49E+07	Comoros	8.535802	Cote d'Ivoire	2.64E+08	Timor-Leste	22.28513
Lesotho	6.68E+07	Honduras	8.556885	Indonesia	2.78E+08	Solomon Islands	22.4044
Swaziland	6.80E+07	Burkina Faso	8.836448	Vietnam	2.84E+08	Kenya	25.0509
Lao PDR	7.20E+07	Congo, Dem. Rep.	9.466156	Ghana	2.87E+08	Zimbabwe	25.35398
Honduras	7.54E+07	Bosnia and Herzegovina	9.725767	Haiti	2.91E+08	Kiribati	25.46933
Nicaragua	7.86E+07	Kyrgyz Republic	10.14364	Pakistan	3.89E+08	Malawi	27.11284
Mexico	7.95E+07	Central African Republic	10.56082	Zimbabwe	3.91E+08	Haiti	27.56047
Chad	8.04E+07	Ghana	10.63635	Rwanda	4.05E+08	Sierra Leone	29.69332
Guatemala	8.60E+07	Jordan	10.78265	Malawi	4.63E+08	Lesotho	31.13248
Jordan	9.50E+07	Mongolia	10.79306	Bangladesh	5.31E+08	Cabo Verde	32.0282
Botswana	9.52E+07	Lao PDR	10.94937	Afghanistan	5.40E+08	Mozambique	32.20256
Thailand	1.05E+08	Ethiopia	10.96349	Zambia	5.87E+08	Tonga	34.1797
Ukraine	1.08E+08	Dominican Republic	11.59233	Congo, Dem. Rep.	6.98E+08	Rwanda	35.72102
Angola	1.17E+08	Cote d'Ivoire	11.70299	India	7.14E+08	Zambia	37.60197
Dominican Republic	1.21E+08	Guinea-Bissau	12.50278	Uganda	8.24E+08	Botswana	43.91529
Namibia	1.26E+08	Djibouti	12.80861	Colombia	8.25E+08	Sao T. & Principe	50.61343
Burundi	1.30E+08	Nicaragua	13.06394	Mozambique	8.76E+08	Liberia	50.61999
Cameroon	1.31E+08	Burundi	13.13727	South Africa	8.92E+08	Swaziland	52.52994
Sudan	1.34E+08	Benin	13.16867	Ethiopia	1.07E+09	Namibia	52.9663
Benin	1.35E+08	Cambodia	13.21426	Kenya	1.15E+09	Vanuatu	57.29643
China	1.39E+08	Mali	14.38199	Tanzania	1.16E+09	Micronesia	379.5527
Madagascar	1.39E+08	Moldova	15.27242	Nigeria	1.36E+09		
Niger	1.42E+08	Senegal	15.62616				

STATA commands for within-between regression

```
// "Within-between" estimator (augmented RE estimation)

gen logPOP_demean = logPOP-logPOP_mean

gen logGDPpcUSD_demean = logGDPpcUSD-logGDPpcUSD_mean

gen logDALYR3_demean = logDALYR3-logDALYR3_mean

gen logU5MR_demean = logU5MR-logU5MR_mean

gen logMMR_demean = logMMR-logMMR_mean

gen DTP3_demean = DPT3-logDPT3_mean

gen fertility_demean = fertility-fertility_mean


xtreg logDAHpc logPOP_demean logPOP_mean logGDPpcUSD_demean logGDPpcUSD_mean
logDALYR3_mean logDALYR3_demean logU5MR_mean logU5MR_demean logMMR_mean
logMMR_demean DTP3_mean DTP3_demean fertility_mean fertility_demean i.year, re vce (cluster
CID)
```

References

Belsley, D. A., Kuh, E., & Welsch, R. E. (2005). Detecting Influential Observations and Outliers *Regression Diagnostics* (pp. 6-84): John Wiley & Sons, Inc.
